# Methylene Blue in Refractory Shock

**DOI:** 10.7759/cureus.31158

**Published:** 2022-11-06

**Authors:** Som N Chalise, Taaliba A Sahib, Gregory A Boyer, Vikas Pathak

**Affiliations:** 1 Pulmonary and Critical Care Medicine, Riverside Health System, Newport News, USA; 2 Emergency Medicine, Riverside Health System, Newport News, USA

**Keywords:** nitric oxide-cgmp pathway, overdose, vasoplegia, refractory shock, methylene blue infusion

## Abstract

Many patients suffer shock in intensive care units (ICU). The majority of the patients with shock respond to standard treatment with vasopressors in addition to the treatment of underlying etiology. Some may not respond to vasopressors and have high mortality. To those patients who do not respond, methylene blue has been used in the past with some success. We present a case report on the use of methylene blue along with a brief literature review.

## Introduction

One of the biggest challenges encountered in the intensive care unit (ICU) is shock. Patients who are afflicted with shock for a variety of reasons that generally precede that condition often face a milieu of obstacles prior to any improvement, including multi-organ system failure, among other life-threatening conditions. Despite the availability of several therapeutic options to combat circulatory collapse and its adverse effects on multiple organs, a percentage of patients display no physiological response to traditional solutions. The treatment of shock is primarily the treatment of underlying etiology, for example, to identify the source of infection and treat with antibiotics and remove the source of infection when needed in septic shock. The medications commonly used to support hemodynamics in shock include norepinephrine, epinephrine, vasopressin, phenylephrine, and sometimes dobutamine or milrinone. The purpose of this paper is to introduce a possible solution to distributive shock patients that is demonstrably refractory to conventional methods.

Methylene blue is a compound initially discovered in 1876 and was utilized primarily in the textile industry as a pigmentation agent, gradually becoming instrumental in the manufacturing of phenothiazine antipsychotic agents [1]. Notwithstanding its humble beginnings, methylene blue has been used in critical care units across the country, even spending a brief foray as a therapeutic alternative for malaria treatment [1] and in the treatment of methemoglobinemia. Clinical trials for the efficacy of methylene blue were conducted over a number of years, many of which espousing the utility of the drug in the use of refractory shock. While early concerns of pulmonary adverse effects were noted in clinical trials, the agent was notably attributed to significant increases in mean arterial pressure and simultaneously decreasing catecholamine requirements for septic shock patients [2]. Promising responses to the hemodynamic improvement in septic and distributive shock patients after the administration of methylene blue are hampered by an indeterminate effect in mortality and morbidity [2]. As a result, the official recommendation for its widespread use in critical care units continues to be in question.

## Case presentation

A 20-year-old female with a medical history of postural orthostatic tachycardia syndrome, depression, chronic migraines, and seizures was hospitalized after being found unresponsive. She did not have a history of drug abuse; however, she overdosed on multiple medications with a suicidal note sent to families. Although the actual amount of medications ingested was unclear, the patient was found with empty bottles of hydromorphone, promethazine, hydroxyzine, cyclobenzaprine, and acetaminophen around her. The patient was admitted profoundly hypotensive with a blood pressure of 60/40 mmHg, hypothermic at 34°C, and with agonal breathing, maintaining saturations in the 70s with bag mask valve respirations. She was promptly intubated endotracheally and started on a mechanical ventilator. The patient was in severe metabolic acidemia with a pH of 7.05 (normal value: 7.35-7.45). Her bicarbonate was 10 mmol/L (normal level: 22-32 mmol/L), as well as her lactic acidemia with lactic acid level of 5.8 mmol/L (normal value: less than 2 mmol/L), which worsened to 10.0 with repeat check in a few hours. Her urine for a common toxicology screen resulted positive for opiates, and others were negative. Her serum acetaminophen level was elevated at 555 mcg/mL with a therapeutic level of 10-25 mcg/mL, and her ethanol level was 219 mg/dL. Complete blood count was unremarkable.

The patient was started on N-acetylcysteine, intravenous (IV) crystalloids of 30 cc/kg bolus, and vasopressors therapy. Her acetaminophen level decreased to 184.9 mcg/mL in repeat testing within 12 hours and continued to decline quickly with subsequent testing. Her blood urea nitrogen and creatinine were normal at the time of presentation and stayed normal throughout hospital stay. The patient's condition rapidly got worse, and within 12 hours of presentation, she required the use of four vasopressor agents with minimal improvement in her hemodynamics. Pan-cultures were sent, and she was given empiric broad-spectrum antibiotics with intravenous vancomycin and piperacillin-tazobactam due to bilateral lung infiltrates with concern for aspiration event. Her urinalysis did not show evidence of infection. Her echocardiogram showed normal ejection fraction of 60%, normal right-sided pressure, no pericardial effusion, and normal valvular structure and chamber size. Due to refractory shock despite adequate volume resuscitation and four vasopressors at maximum dosages (norepinephrine at 30 mcg/minute, vasopressin at 0.03 units/minute, epinephrine at 10 mcg/minute, and phenylephrine at 180 mcg/minute), she was given one dose of methylene blue at 2 mg/kg. Over the next 4-8 hours, the patient's hemodynamics started to improve significantly. By the end of eight hours of methylene blue, her vasopressor requirements came down to just norepinephrine at 2 mcg/minute. Her mental status improved, and she started to follow commands. Her lactic acidemia also improved from 10 mmol/L to 4 mmol/L by 24 hours of methylene blue. Her blood culture did not show any growth. Her respiratory culture grew only very scant candida albicans, which was not considered to be an etiology. Due to the aspiration event with bilateral pulmonary infiltrates at the time of presentation, she developed severe acute respiratory distress syndrome (ARDS) within another 48 hours and was unresponsive to conventional treatment requiring transfer to a tertiary care center for extracorporeal membrane oxygenation (ECMO). At the tertiary center, the patient underwent venovenous ECMO for a few days. Her respiratory status improved, and she was successfully extubated on day 9 of intubation. She was later evaluated by psychiatry and admitted to the inpatient psychiatry unit.

## Discussion

Studies have suggested that methylene blue is instrumental in the inhibition of the nitric oxide-cyclic guanosine monophosphate (cGMP) pathways and responsible for systemic vasodilation, in the pathophysiology seen in septic and anaphylactic shock [3]. After conventional methods regarding the treatment of distributive shock, including intravenous fluids and vasopressor therapies, are unsuccessful, disrupting the nitric oxide mechanism of vasodilation may be a feasible alternative to avoid or reverse post-shock complications. The loss of systemic vascular resistance typically characterized in distributive shock physiology is the factor that methylene blue counters with varying levels of success [3]. In anaphylaxis, specifically, however, methylene blue appears to be responsible for the inhibition of the soluble protein guanylyl cyclase, which itself is instrumental in vasodilation instigated by histamine [3]. Case reports have also shown methylene blue to be useful in vasoplegia following post-cardiopulmonary bypass or after dihydropyridine toxicities [3]. The relative safety of methylene blue in refractory shock may encourage its usage in critical care arenas and improve the outcome of patients with distributive shock presentations.

Our patient presented with drug overdose with possibly multiple substances. Alcohol and acetaminophen levels were high, and opiate was detected on the toxicology screen. Acetaminophen level quickly started to decline with N-acetylcysteine therapy. She had pulmonary infiltrates from an aspiration event; otherwise, infections were ruled out. She was in refractory shock requiring four vasopressors. When a single dose of methylene blue was administered, her hemodynamics improved significantly within 4-8 hours. It is likely the response to methylene blue. There have been published case reports and case series for the use of methylene blue in the treatment of refractory shock. From its premiere study in a randomized trial in 2001, methylene blue has shown tremendous strides toward being represented as a mainstay in distributive shock treatment [3]. The improvement of overall hemodynamics and the resulting increased tissue perfusion, resulting in decreases in end-organ damage, have only since been hindered by the paucity of confirmatory studies that validate the diminishing mortality and morbidity outcomes [4]. The studies do confirm the overall reduction of concurrent adrenergic support use and the countering of the myocardial depression often seen in distributive shock patients [4].

Further details regarding these studies are summarized in Table 1 and have been well documented over the past 25 years. Since the death rate of septic shock is very high (up to 50%) [5], even a small benefit from methylene blue would be important for these patients. While the conclusion regarding methylene blue in distributive shock cases has shown promise in the last 25 years, its application and utilization parameters remain nebulous. Support for the future use of methylene blue in critical care patients continues to grow as the applications regarding amelioration of hemodynamic instability have consistently warranted its use. Understanding the pathophysiology surrounding distributive shock and its connection to nitric oxide vasodilation pathways is essential to the utilization of methylene blue in critical care cases involving refractory shock. Critical care arenas that elect to incorporate methylene blue in septic shock pathways may represent the next evolution of an agent that, thus far, has continued to prove mercurial in its employment in the medical field.

**Table 1:** Literature review of methylene blue in refractory septic shock n: number of patients studied; N/A: not applicable or not available during our literature search; mg: milligram; mg/kg: milligram/kilogram; mL: milliliter; PaO2: partial pressure of oxygen in mmHg; FiO2: fraction of inhaled oxygen; ICU: intensive care unit; IV: intravenous

Study	Study Design	Study Size (n)	Patient Presentation	Intervention Versus Control	Outcomes	Morbidity/Mortality Outcomes
Porizka et al., 2020 [6]	Retrospective analysis	20	Refractory distributive shock and medical and surgical patients	Responders (45%) versus non-responders (55%) to single-dose methylene blue	Profound tissue hypoxia and high anaerobic metabolism were associated with decreased hemodynamic responsiveness to methylene blue administration	Lower 24-hour mortality (0% versus 45.5%, P=0.038) and lower ICU/30-day mortality (44.4% versus 100%, P=0.008) of responders versus non-responders, respectively
Memis et al., 2002 [7]	Prospective, randomized, double-blinded, and placebo-controlled	30	Severe sepsis and bacterial infections	0.5 mg/kg/hour methylene blue diluted in 100 mL of isotonic saline over six hours versus 100 mL of isotonic saline over six hours	No significant effect on plasma cytokine levels, blood gases, or biochemical parameters. Methylene blue did result in significantly higher mean arterial pressures and methemoglobin levels when compared to control and baseline levels. No mortality difference between methylene blue and controls	No significant difference in mortality rates (26.6%), ventilation duration (P>0.05), ventilation-free days (P>0.05), or ICU length of stay (P>0.05)
Andresen et al., 1998 [8]	Prospective sequential study and consecutive patients	10	Severe septic shock, various causes, and two vasopressors	1 mg/kg IV bolus of methylene blue	Significant increases in systolic, diastolic, and mean arterial pressures. Significant increase in systemic vascular resistance. Unchanged cardiac output, oxygen consumption, or oxygen extraction ratio	N/A
Brown et al., 1996 [9]	Case report	1	Septic shock, pneumonia, norepinephrine, and dopamine	Five 100 mg doses of methylene blue followed by continuous infusion over 44 hours	Improved blood pressure and decreased vasopressor requirements	N/A
Kirov et al., 2001 [4]	Prospective, randomized, controlled, and open-label	20	Septic shock	2 mg/kg bolus methylene blue followed by infusion versus isotonic saline	Reduced norepinephrine, epinephrine, and dopamine requirements. Maintained oxygen transport	Five treated with methylene blue survived versus three treated with conventional treatment. No significance measured
Weingartner et al., 1999 [10]	Prospective, open, and non-randomized clinical trial	10	Severe septic shock requiring vasopressors and mechanical ventilation	One hour infusion of 4 mg/kg methylene blue	Increase in mean arterial pressure and systemic vascular resistance index and increase in left ventricular stroke work index. Increase in pulmonary vascular tone and decrease in gas exchange	N/A
Park et al., 2005 [11]	Prospective, open, and consecutive patients	20	Septic shock refractory to dopamine infusion >20 mcg/kg/minute	1 mg/kg methylene blue infusion	Increased mean arterial pressure without a decrease in cardiac output. Increase pulmonary vascular resistance. However, no change in gas exchange	54.5% of responders survived when compared with 11.1% of non-responders. However, no clinical significance (P=0.07)
Gachot et al., 1995 [12]	Prospective, open, and single-dose study	6	Severe septic shock	3 mg/kg methylene blue infusion	Increased systemic and pulmonary vasoconstrictive and no significant decrease in cardiac index. Decreased PaO2/FiO2 ratio without significant modification of intrapulmonary shunting	One survived. No significance measured
Grayling and Deakin, 2003 [13]	Case study	1	Septic shock, infective endocarditis refractory to dopamine, norepinephrine, and phenylephrine	2 mg/kg methylene blue preoperatively and 1 mg/kg/hour infusion intraoperatively during cardiopulmonary bypass	Phenylephrine and norepinephrine were discontinued. Maintained intraoperatively on dopamine and methylene blue with maintained blood pressure goals. Decreased PaO2/FiO2 ratio noted postoperatively	N/A
Daemen-Gubbels et al., 1995 [14]	Open-label, non-randomized clinical trial and consecutive patients	9	Septic shock	2 mg/kg methylene blue infusion over 20 minutes	Increased mean arterial pressure and oxygen uptake, decreased arterial compliance, increased myocardial function, and oxygen delivery	N/A

## Conclusions

Our case responded well to IV methylene blue therapy when the patient was in refractory shock. Further data will be needed to conclude whether methylene blue is effective consistently and if it has any value as a mortality benefit in distributive shock patients.
